# To be or not to be a fat burner, that is the question for cpt1c in cancer cells

**DOI:** 10.1038/s41419-023-05599-1

**Published:** 2023-01-24

**Authors:** Rut Fadó, Sebastian Zagmutt, Laura Herrero, Helena Muley, Rosalía Rodríguez-Rodríguez, Huichang Bi, Dolors Serra, Núria Casals

**Affiliations:** 1 Basic Sciences Department, Faculty of Medicine and Health Sciences, Universitat Internacional de Catalunya, E-08195 Sant Cugat del Vallès, Spain; 2 Institut de Neurociències, Universitat Autònoma de Barcelona, E-08193 Bellaterra, Cerdanyola del Vallès, Spain; 3 Department of Biochemistry and Physiology, School of Pharmacy and Food Sciences, Universitat de Barcelona, E-08028 Barcelona, Spain; 4 Institut de Biomedicina de la Universitat de Barcelona (IBUB), Universitat de Barcelona, E-08028 Barcelona, Spain; 5 Centro de Investigación Biomédica en Red de Fisiopatología de la Obesidad y la Nutrición (CIBEROBN), Instituto de Salud Carlos III, Madrid, Spain; 6 School of Pharmaceutical Sciences, Southern Medical University, Guangzhou, 510515 China

**Keywords:** Cancer metabolism, Membrane proteins, Prognostic markers

## Abstract

There is an urgent need to identify reliable genetic biomarkers for accurate diagnosis, prognosis, and treatment of different tumor types. Described as a prognostic marker for many tumors is the neuronal protein carnitine palmitoyltransferase 1 C (CPT1C). Several studies report that CPT1C is involved in cancer cell adaptation to nutrient depletion and hypoxia. However, the molecular role played by CPT1C in cancer cells is controversial. Most published studies assume that, like canonical CPT1 isoforms, CPT1C is a mediator of fatty acid transport to mitochondria for beta-oxidation, despite the fact that CPT1C has inefficient catalytic activity and is located in the endoplasmic reticulum. In this review, we collate existing evidence on CPT1C in neurons, showing that CPT1C is a sensor of nutrients that interacts with and regulates other proteins involved in lipid metabolism and transport, lysosome motility, and the secretory pathway. We argue, therefore, that CPT1C expression in cancer cells is not a direct regulator of fat burn, but rather is a regulator of lipid metabolic reprograming and cell adaptation to environmental stressors. We also review the clinical relevance of CPT1C as a prognostic indicator and its contribution to tumor growth, cancer invasiveness, and cell senescence. This new and integrated vision of CPT1C function can help better understand the metabolic plasticity of cancer cells and improve the design of therapeutic strategies.

## Facts

- CPT1C favors tumor survival in conditions of hypoxia and nutrient deprivation and is a prognostic marker in several human tumors.
- In the cancer field, CPT1C is considered a marker of fatty acid oxidation, which is controversial because it has inefficient catalytic activity, and is located in the endoplasmic reticulum of cells.
- Evidence from the field of neuroscience allow us to deduce that CPT1C is a nutrient sensor that regulates the function of other proteins involved in lipid metabolic reprogramming.

## Open questions

- Identify the molecular mechanism by which CPT1C modulates mitochondrial function.
- Only 3 proteins are known to date to interact with CPT1C and be regulated by CPT1C sensing of nutrients. What other proteins relevant to tumor growth bind CPT1C?
- The crystal structure of CPT1 proteins, and the design of new drugs that specifically target the CPT1C isoform.

## Introduction

Metabolic reprograming is a signature of cancer cells that allows them to adapt to environmental stresses such as hypoxia and nutrient scarcity [1]. Currently, there is a search for surrogate genetic markers involved in metabolic reprograming that would lead to more accurate diagnosis and prognosis, but also optimized targeted therapy [1]. Studies have shown that lipid metabolism alterations are associated with tumor growth and progression [2, 3]. Indeed, some cancer cells exhibit a high rate of fatty acid oxidation (FAO) in producing energy [4, 5], and identified as emerging therapeutic targets are key genes involved in FAO, such as carnitine palmitoyltransferase 1 (CPT1) [6, 7].

CPT1 enzymes catalyze the conversion of long-chain acyl-CoAs to acyl-carnitines to facilitate fatty acid (FA) entry to the mitochondrial matrix for their beta-oxidation [8, 9]. The canonical members of the CPT1 family are CPT1A, a ubiquitous enzyme with greater expression in liver, pancreas, and kidney, and CPT1B, expressed mainly in muscle, heart, and brown fat tissue. Canonical CPT1 enzymes control the limiting step in FAO, and their activity is regulated by malonyl-CoA, a metabolite mainly derived from glucose and a precursor of FA synthesis. In the feeding state, CPT1A and CPT1B are inhibited by malonyl-CoA, whose levels increase when the energy balance is positive. By contrast, in conditions of glucose deprivation, malonyl-CoA levels decrease, leading to the activation of canonical CPT1 enzymes, and driving FAO and ATP production [8, 9]. Therefore, FAO is finely regulated, depending on nutritional state, through the malonyl-CoA-CPT1A/B axis.

CPT1C is an intriguing isoform: despite its high sequence similarity with CPT1A and CPT1B, it has hardly any catalytic activity and is mainly localized in the endoplasmic reticulum (ER) rather than in mitochondria [10–12], indicating a cellular role different from that of the canonical CPT1 enzymes. CPT1C is expressed almost exclusively in neurons, stem cells, and cancer cells [12–14]. Interestingly, CPT1C maintains the ability to bind malonyl-CoA, and therefore, to sense the nutritional status of the cells [10, 11]. Our group has recently demonstrated that the main role of CPT1C in neurons is to regulate the activity of other proteins, with which it forms complexes, depending on malonyl-CoA levels [15–17]. Specifically, those other proteins are involved in the regulation of lipid metabolism, Golgi-mediated secretory transport, and the endolysosomal system, impacting on synaptic transmission and axon growth [15–19]. Therefore, in neurons, CPT1C is a nutrient sensor that regulates neuron metabolism and function.

However, in the cancer field, most authors assume that CPT1C has the same catalytic function as the other canonical members of its enzyme family, and that CPT1C is a specific marker of FAO. This assumption comes from studies demonstrating that CPT1C overexpression and silencing regulates FAO [14, 20–22]. Here, we exhaustively review the literature and question the role of CPT1C as a facilitator of FA entry in the mitochondria of cancer cells. Instead, we propose that CPT1C is a sensor of cell nutritional status and a hallmark of lipid metabolic adaptation.

### CPT1C in human tumors

The first time that CPT1C was associated with tumor growth was in 2011 [14], 9 years after its discovery as a new CPT1 isoform [10]. Searching for transcripts involved in tumor cell metabolic transformation, Zaugg and collaborators [14] found that 2 independent screening methods unexpectedly concurred in pointing to the *Cpt1c* gene. *Cpt1c* was defined as a new cancer gene that both conferred rapamycin resistance and was activated by p53. The same authors then analyzed CPT1C expression in patients with non-small cell lung carcinoma, finding that, CPT1C mRNA was upregulated in 81% of cases of tumorous tissues compared to normal adjacent tissue; interestingly, they also found that CPT1C was expressed in a broad range of human malignancies and suggested that CPT1C was involved in a parallel pathway to mTORC-enhanced glycolysis [23].

**Fig. 1:**
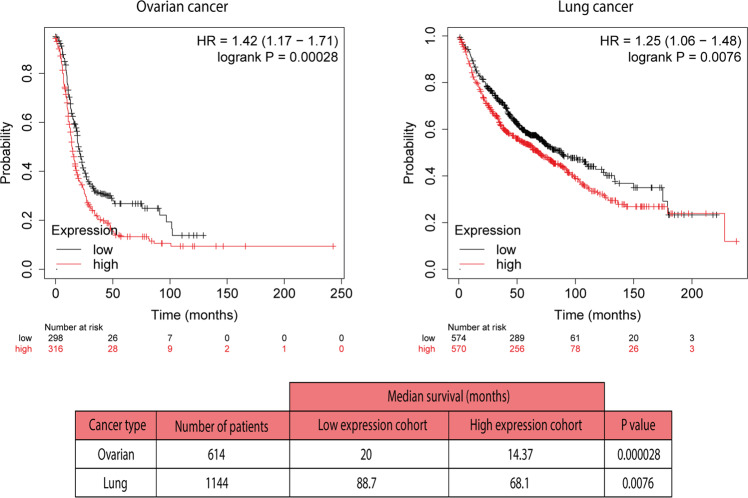
Kaplan–Meier analysis. Overall survival rate for patients expressing low or high CPT1C mRNA levels (614 and 1144 patients with ovarian cancer and lung cancer, respectively) analyzed with Kaplan–Meier plotter. Dataset and analysis system from [62].

Other authors subsequently reported higher CPT1C expression (mRNA or protein levels) in other types of tumors, e.g., papillary thyroid carcinomas [24] and hepatocellular carcinomas [21] than in normal tissues. Moreover, patients with gastric cancer, hepatocarcinoma, and basal-like breast cancer with high CPT1C expression were reported to experience significantly shorter overall survival compared to patients with low CPT1C expression [20–22]. Kaplan-Meier analysis confirms the same poor survival in patients with ovarian or lung cancer with high CPT1C expression (Fig. 1), pointing to CPT1C as a prognostic marker for several tumor types. Additionally, a study using The Cancer Genome Atlas (TCGA) dataset showed that CPT1C is part of the 5-gene metabolic signature associated with epithelial-mesenchymal transition, metastatic progression, and poor prognosis across multiple cancers [25].

However, not all tumor types benefit from CPT1C expression, since human bladder cancer samples compared to normal adjacent tissues show decreased levels of CPT1C. [26].

### CPT1C favors tumor survival and growth in response to stress

Numerous reports demonstrate the key role of CPT1C in human-derived cancer cell lines in response to metabolic stress. CPT1C overexpression in breast cancer (MCF7) and papillary thyroid carcinoma (KTC-1) cell lines has been reported to promote survival in response to hypoxia or glucose depletion [14, 24]. Accordingly, CPT1C downregulation by siRNAs in colon cancer (HCT116), breast cancer (MCF7, Hs578T), lung carcinoma (A549), pancreatic carcinoma (PANC-1), and thyroid carcinoma (KTC-1 and B-CPAP) cell lines increases cell death in response to different stressors, such as hypoxia, glucose depletion, glycolysis inhibition, and rapamycin treatment (which mimics amino acid-like starvation) [14, 24, 27]. Overall, those data indicate that CPT1C expression provides some metabolic advantage that favors tumor growth in the face of different metabolic challenges. This feature may be of high relevance for cells in the central zone of solid tumors, which are usually poorly irrigated and have low access to nutrients and oxygen. The fact that blood tumor cell lines are those with less CPT1C expression may reflect their easy availability to nutrients in the bloodstream [23].

**Fig. 2:**
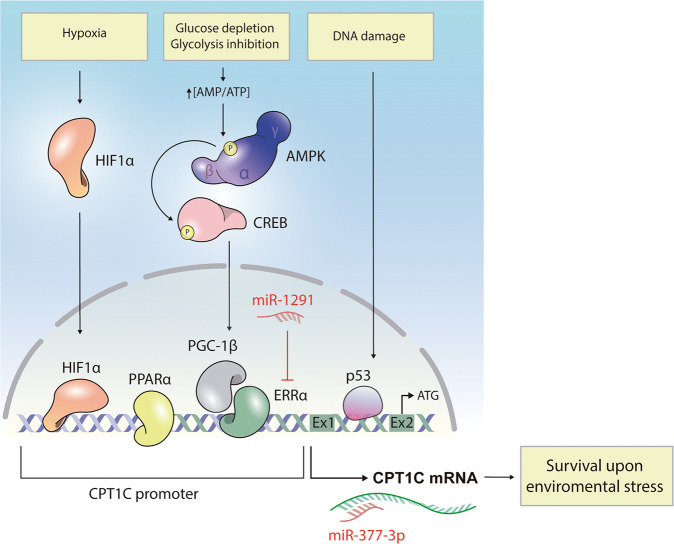
Regulation of CPT1C expression. Metabolic challenges such as hypoxia and nutrient deprivation increase CPT1C expression in most cancer cells, promoting metabolic adaptation and survival in response to these environmental stressors. On the one hand, low oxygen levels promote accumulation of the HIF1α transcription factor, which is translocated to the nucleus to bind the *Cpt1c* promoter and activate gene transcription. On the other hand, glucose depletion and glycolysis inhibition increase the AMP/ATP ratio leading to activation of AMPK, which in turn, phosphorylates and activates the CREB transcription factor. Phosphorylated CREB increases expression of the PGC-1β and ERRα transcription factors, ultimately promoting *Cpt1c* transcription. PPARα activates CPT1C expression by binding directly to the promoter region. p53 is activated by DNA-damaging stimuli and binds to the first intron of the *Cpt1c*, thereby enhancing CPT1C expression. Interestingly, 2 different miRNAs have been described that downregulate this signaling pathway, one acting on ERRα expression, and the other on CPT1C expression.

Interestingly, and in accordance with its role in cell metabolism, CPT1C transcription is precisely regulated in response to distinct metabolic challenges (Fig. 2). On the one hand, oxygen restriction (0.2%) increases CPT1C transcription through binding of the hypoxia-inducible factor HIF1α to the *Cpt1c* promoter [20]. Moreover, tumor-bearing mice subjected to chronic hypoxia showed an increase in CPT1C expression in the tumor mass [14]. Interestingly, this increase in CPT1C expression was not found in the normal adjacent tissue, suggesting that hypoxic regulation of CPT1C depends on the unique circumstances of the tumor microenvironment.

On the other hand, glucose depletion or glycolysis inhibition by 2-deoxy-D-glucose also increase CPT1C mRNA levels in different cancer cell lines [24, 28]. Wu and collaborators [28] moreover, elegantly demonstrated that this regulation is mediated by the energy sensor AMPK (which is phosphorylated and activated when ATP levels decrease) through the downstream signaling axis CREB-PGC-1β-ERRα [28]. The proteins PGC-1β and ERRα form functional complexes to regulate the expression of genes involved in long-term and sustained adaptive responses to environmental stressors, such as nutrient deprivation [29]. The *Cpt1c* promoter has multiple ERRα binding sites that effectively promote *Cpt1c* transcriptional activation, as demonstrated by dual luciferase reporter gene assays and ChIP assays [30]. Interestingly, mouse embryonic fibroblasts deficient for AMPK fail to upregulate CPT1C when exposed to low-glucose and/or hypoxic conditions [14], confirming that CPT1C expression in response to energy stress is controlled by AMPK.

Recently, Bi’s group [31] has discovered that PPARα, a ligand-activated transcription factor that regulates lipid metabolism and tumor progression, also activates CPT1C expression by binding directly to the promoter region of CPT1C, indicating that CPT1C is a novel downstream target gene of PPARα [31]. Drug treatments that affect cellular metabolism, such as metformin (an AMPK activator) and imatinib (a glycolysis inhibitor), also upregulate CPT1C expression [14, 32]. Finally, note that CPT1C is a bona fide target of the tumor suppressor p53, and therefore, CPT1C expression is enhanced by DNA-damaging stimuli, such as irradiation and certain chemotherapeutic drugs [33]. Interestingly p53 is also activated by stress stimuli such as hypoxia and starvation, having a key role in metabolic reprogramming [33]. It has recently been demonstrated that the mutated form of p53, which is prevalent in human cancer, maintain the ability of activating CPT1C expression in basal-like breast cancers through the miR-200c-ZEB2 axis [22]. However, in human non-small-cell lung carcinoma, CPT1C provided a growth advantage that did not correlate with p53 expression or mutation status, indicating that CPT1C is also involved in a p53-independent metabolic adaptation [14].

In summary, it seems that CPT1C upregulation is involved in enabling cellular adaptation to ever-changing metabolic conditions in tumor cells (Fig. 2).

### CPT1C involvement in migration, invasion, and cancer cell senescence

**Fig. 3:**
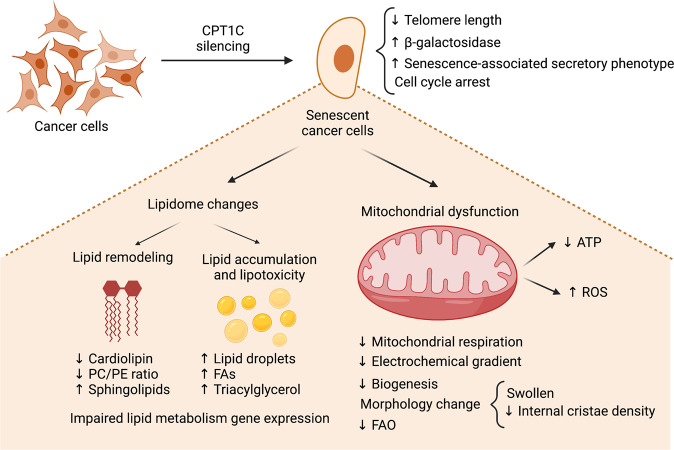
Effects of CPT1C silencing in cancer cell metabolism. Downregulation of CPT1C favors cancer cell senescence, as demonstrated by the increased b-galactosidase marker, diminished telomere length, cell cycle arrest, and activation of the senescence-associated secretory phenotype. CPT1C downregulation, as revealed by lipidomic analysis, results in great lipid remodeling, including impaired expression of lipid metabolic genes, decreased cardiolipin levels, diminished phosphatidylcholine/phosphatidylethanolamine (PC/PE) ratio, increased levels of FAs, triacylglycerol, and sphingolipids, and lipid droplet accumulation. CPT1C downregulation impairs mitochondrial morphology, function (decreased mitochondrial respiration, decreased FAO, and altered electrochemical gradient) and biogenesis. All those changes result in decreased ATP levels and oxidative stress.

In addition to its role in meeting metabolic challenges, CPT1C also protects cells from senescence (Fig. 3). Late PANC-1 cells (with many passages) or PANC-1 cells stably transfected with an empty vector show a myriad of characteristics that reflect cell senescence, such as increased beta-galactosidase staining, cell morphology changes, cell cycle arrest, decreased telomere elongation, and reduced gene expression of the senescence-associated secretory phenotype [27, 34]. The senescence phenotype includes impairment of mitochondrial function, reduced cellular ATP levels, increased ROS accumulation, and IL-8 inflammation marker expression. Interestingly, CPT1C overexpression in senescent cells can reverse the phenotype, while CPT1C downregulation recapitulates senescence in diverse cancer cell lines, leading to lower proliferation, migration, and invasion rates, and reduced in vivo tumor growth [14, 20, 21, 27]. Interestingly, of all the members of the CPT family, CPT1C exhibits the most substantial effect on cell senescence and mitochondrial dysfunction [35].

The effect of CPT1C on cancer cell senescence described above is also present in normal diploid fibroblast MRC-5 cells [36]. Moreover, CPT1C expression in a non-tumorigenic epithelial cell line (MCF12A) enhances epithelial-mesenchymal transition phenotypes, such as migration, invasion, and anchorage-independent growth [22].

It is worth mentioning that 2 miRNAs with antitumor roles have been described that downregulate CPT1C expression and cause cell senescence (Fig. 2). First, miR-377-3p directly targets the CPT1C 3’-untranslated region and decreases proliferation, migration, and invasion of hepatocarcinoma cells [21]. Accordingly, in patient hepatocarcinoma samples, miR-377-3p levels are inversely correlated with CPT1C expression and predict clinical outcomes. Second, miR-1291 downregulates CPT1C expression by targeting the transcription factor ERRα [30]. Consequently, cell proliferation is decreased, and lipid metabolism is impaired. Targeting these miRNAs has been proposed as a therapeutic strategy to combat malignant cancers with high CPT1C expression [21, 23, 30, 37].

### What metabolic pathways are regulated by CPT1C?

CPT1C silencing in different cancer cell lines leads to important alterations in mitochondrial function and morphology: loss of mitochondrial electrochemical gradient [27, 35], reduced mitochondrial respiration (basal oxygen consumption rate and maximal respiration using glucose as a substrate) [27, 35], reduced levels of the mitochondrial-specific lipid cardiolipin [35, 38], swollen mitochondria with loss of internal crista density, and abnormal membrane structures [14, 27] (Fig. 3). All these changes are associated with an increase in ROS formation [27, 35, 38] and a decrease in ATP production [14, 21, 27, 35]. Moreover, genes involved in the electron transport chain and mitochondrial biogenesis are downregulated [27, 35, 38] (Fig. 3). Interestingly, CPT1C has also been involved in neuron redox homeostasis system. Specifically, an increase in the glutathione oxidized form (GSSG) was detected in CPT1C KO mouse brains resulting in an increased oxidative environment and a reduced capacity to protect cells from oxidative damage [39]. Accordingly, CPT1C overexpression in a hippocampal cell line downregulated ROS and malondialdehyde (a final product of polyunsaturated fatty acid peroxidation) and increased *superoxide dismutase* (an important antioxidant mechanism) in response to cell damage [40]. All that evidence points to the idea that CPT1C is necessary to maintain normal mitochondrial performance in cancer cells.

The lipidomic profile is also altered by CPT1C silencing (Fig. 3), as an increase in total FA, triacylglycerol, and sphingomyelin content and an accumulation of lipid droplets have been reported [21, 35, 38]. Moreover, an increase in cell membrane stiffness and permeability is suggested by a reduced phosphatidylcholine/phosphatidylethanolamine (PC/PE) ratio [38]. Also reduced is the expression of lipid anabolic enzymes, such as DGAT1/2, and FASN, and the transcription factor SREBF1. Interestingly, CPT1C silencing in MDA-MB-231 and PANC-1 cells exerts more significant effects on the mitochondrial function and lipidome than downregulation of *CPT1A* and *CPT1B* [35]. Overall, those data point to an important role for CPT1C in mitochondrial function and in lipid metabolism in cancer cells that goes beyond FAO modulation (Fig. 3).

### The main function of CPT1C is not to burn fat

CPT1C can exert a myriad of metabolic effects, but what is the molecular function of this protein? In the cancer field it is assumed that the function of CPT1C—like that of the other canonical CPT1 proteins—is to facilitate FA transport to the mitochondrial matrix to boost FAO. This idea arises from the very first study linking CPT1C to cancer, which reported that CPT1C overexpression increases FAO in MCF7 [14]. Subsequent studies have reported FAO changes resulting from CPT1C overexpression or silencing in other cancer cell lines [21, 22, 30], even though none of those studies measured the catalytic activity of the protein.

**Fig. 4:**
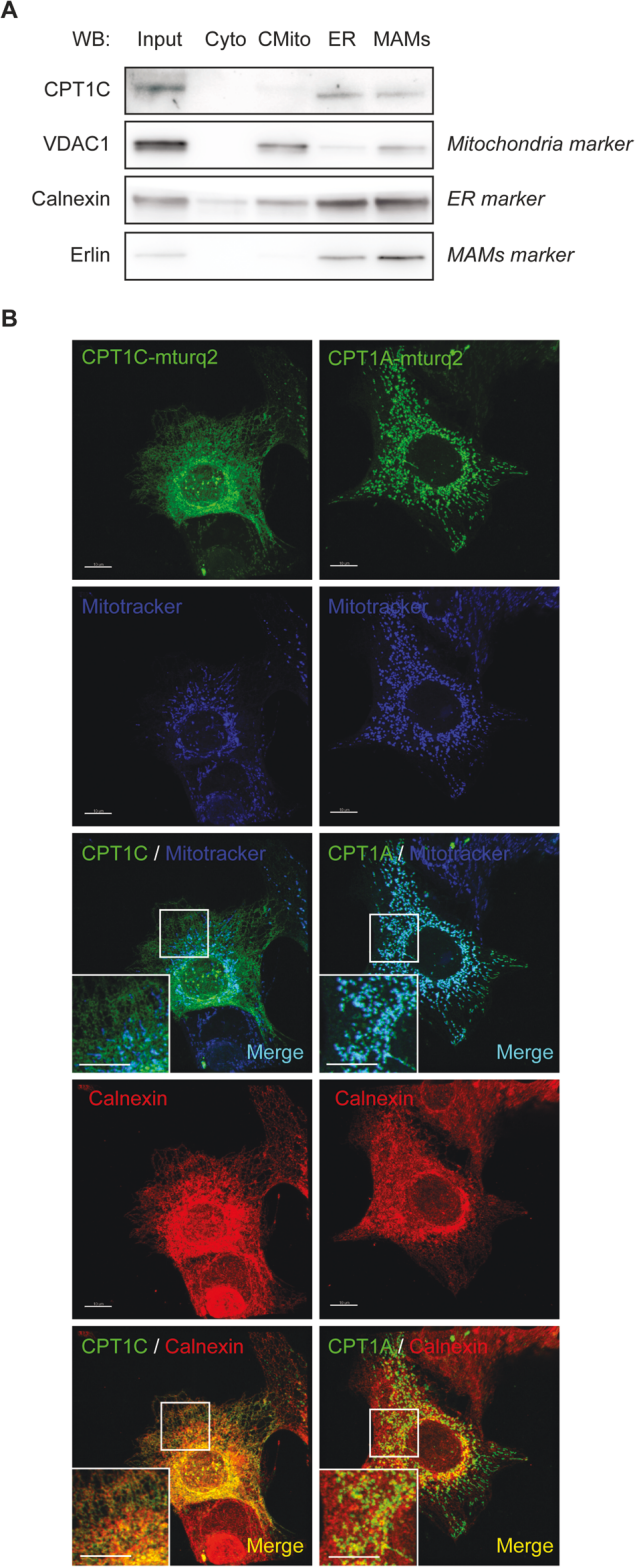
Human CPT1C is mainly localized in ER and mitochondria–ER contact sites in cancer cells. **A** MCF7 cells were seeded, collected and isolated by cellular subfractionation and Percol gradient in order to separate cytoplasm (Cyto), crude mitochondria (CMito, mitochondria and other contaminating membranes), microsomes (fraction containing the ER), and mitochondria-associated membranes (MAMs, fraction containing mitochondria–ER contacts). Also kept was whole lysate (input). All fractions were prepared for the same total protein concentration and processed by Western blotting, and equal volumes of each fraction were loaded. Endogenous CPT1C was mainly localized at ER and MAMs. **B** MCF7 cells were transfected with human CPT1C or CPT1A tagged with mTurquoise2 (mTurq2); 48 h later, Mitotracker Orange (200 nM) was added, and incubation was 30 min. The cells were then fixed. and calnexin (used as an ER marker) was detected by immunocytochemistry. CPT1C mainly colocalized with the ER (orange spots), while CPT1A colocalized with mitochondria (light blue spots). The colocalization of CPT1C with mitochondria (the few light blue spots) may correspond to ER–mitochondria contacts. The scale bar in **B** is 10 µm.
- The fact that CPT1C expression in cancer cells increases with oxygen depletion [14, 20, 24] does not fit with the idea that CPT1C exclusively upregulates FAO. In conditions of hypoxia, cells enhance anaerobic glycolysis over FAO [41]. In fact, ATP generation derived from FADH2 and NADH formed during beta-oxidation requires oxygen. Accordingly, enhanced levels of HIF1α, which plays a fundamental role in cancer hypoxia adaptation driving transition from oxidative to glycolytic metabolism [41–43] correlate with lower expression of FAO-related genes [44], but activates CPT1C expression [20]. All these data suggest that enhanced expression of CPT1C by HIF-1α might support tumor progression by other ways nonrelated to FAO.
- As explained above, CPT1C expression affects the whole mitochondrial function in cancer cells (glucose-mediated respiration, morphology, electrochemical gradient of the membrane, etc) [27, 35, 38]. Therefore, FAO alterations observed in CPT1C silencing may be a consequence of global mitochondrial function changes rather than a direct effect on FA transport to mitochondria.
- CPT1C is involved in proliferation, migration, and invasion of cancer cells in conditions of full glucose availability [14, 20–22, 27].

The following evidence leads us to reason that CPT1C is not a direct regulator of FA entry in mitochondria:

- CPT1C overexpression in yeast and some mammalian tumor and embryonic cell lines (PC12 and HEK293) demonstrates that CPT1C does not have efficient CPT1 activity; specifically, it cannot efficiently convert acyl-CoAs into acyl-carnitines, as the limiting step in FAO [10, 11].
- The assumption that CPT1C is located in the mitochondrial external membrane of cancer cells is still a controversial assumption. We accordingly analyzed CPT1C localization in MCF7 cells by cell sub-fractionation and by confocal microscopy using specific markers for ER and mitochondria. On the one hand, we found that endogenous CPT1C is present in microsomes and mitochondria-associated membranes but is hardly observed in crude mitochondria (Fig. 4A and Supplemental data). On the other hand, we found that CPT1C fused to a fluorescent protein colocalizes mainly with ER and, to a lesser extent, with mitochondria (Fig. 4B). Those findings would indicate that CPT1C is an ER-resident protein present on the ER side of mitochondria–ER contacts, corroborating published data regarding neurons and stem cells [12, 13].

Altogether, those findings lead us to conclude that CPT1C, more than just an enzyme that facilitates FA transport to the mitochondria for FAO, plays a more general role in cancer cells (see Fig. 3).

### CPT1C is a nutrient sensor

Before rethinking the role of CPT1C in cancer cells, it is worthwhile reviewing its function in neurons. Our group has recently demonstrated that CPT1C in neurons acts as a nutrient sensor that regulates the function of other proteins involved in cell metabolism [9]. Specifically, CPT1C regulates the activity of its interacting proteins depending on malonyl-CoA, whose levels recapitulate nutrient availability. Importantly, malonyl-CoA levels increase when the energy balance is positive, and decrease when nutrients are scarce [45]. MNR studies demonstrate that the 5‘ tail of CPT1 switches between 2 conformations—open and closed—depending on malonyl-CoA binding [46, 47]. Therefore, we hypothesize that malonyl-CoA binding triggers a conformational change in CPT1C that affects the activity of its interacting proteins.

**Fig. 5:**
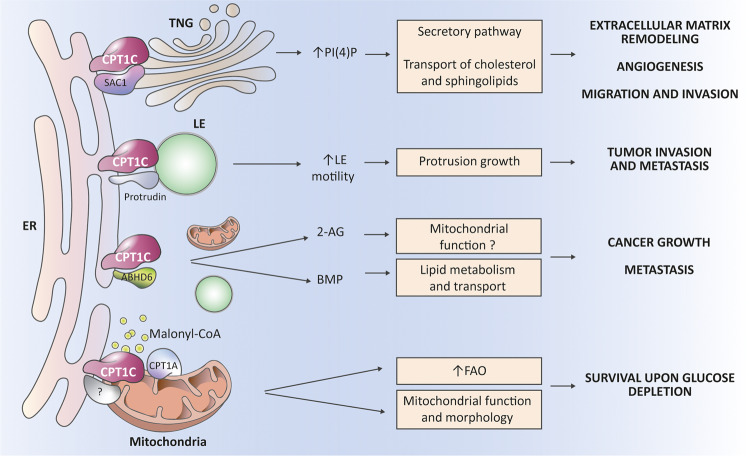
CPT1C regulates the function of key proteins involved in cancer growth and metastasis. CPT1C has inefficient catalytic activity but is able to interact with and regulate the function of other proteins. At the membrane contact sites between the ER and the trans-Golgi network (TGN), CPT1C downregulates the activity of the phosphatase SAC1, favoring the increased PI4P levels necessary for the secretory pathway, and consequently, for remodeling of the extracellular matrix, angiogenesis, and cancer cell migration and invasion. Moreover, PI4P at TGN is necessary for efficient transport of cholesterol and sphingolipids to the plasma membrane, and so can contribute to those processes. At the ER-late endosome (LE) contacts, CPT1C enhances the function of protrudin, which is necessary for anterograde LE transport and for protrusion growth, and therefore, for tumor invasion and metastasis. CPT1C also interacts with the hydrolase ABHD6 and downregulates its activity. This downregulation of ABHD6 increases levels of the endocannabinoid 2-araquidonoylglicerol (2-AG), which, through the CB1 receptor, possibly regulates mitochondrial function. ABHD6 also controls levels of the bis(monoacylglycero)phosphate (BMP) lipid, which is enriched in the intraluminal vesicles of LE and is involved in lipid metabolism and transport. ABHD6 has been associated with tumor growth and metastasis. Finally, we postulate that CPT1C at mitochondria–ER contacts may interact with and regulate some unknown mitochondrial protein triggering changes in mitochondrial function, and additionally postulate that CPT1C locally sequesters malonyl-CoA, and consequently, increases CPT1A activity and FA oxidation.
- Protrudin, which is present in ER-late endosome contact sites, regulates the anterograde transport of late endosomes necessary for axon growth in neurons [51]. In tumors, protrudin orchestrates invadopodia maturation, resulting in tumor cell invasion and metastasis [52, 53]. Interestingly, Palomo–Guerrero and collaborators (17) demonstrate that CPT1C regulates protrudin function depending on malonyl-CoA levels, suggesting that CPT1C can modulate tumor invasion depending on nutrient availability (Fig. 5).
- ABHD6, a hydrolase of monoacylglycerols, regulates levels of the most abundant endocannabinoid, 2-araquidonoylglycerol, which is a modulator of neurotransmitter release through its binding to the endocannabinoid receptor CB1 at the neuron presynaptic membrane [54]. Importantly, CB1 has also been found in the external mitochondrial membrane, where it directly controls mitochondrial respiration and energy production [55]. ABHD6 also hydrolyzes bis(monoacylglycero)phosphate a key lipid for endolysosomal lipid sorting [56]. Interestingly, ABHD6 has been associated with cancer growth and metastasis in different tumor types [57–59]. Since CPT1C upregulates ABHD6 activity when malonyl-CoA levels decrease [17], we postulate that 2-araquidonoylglycerol (2-AG) and bis(monoacylglycero)phosphate (BMP) levels will decrease with nutrient scarcity, resulting in enhanced mitochondrial function and the endolysosomal-mediated lipid transport changes necessary for the metabolic adaptation of cancer cells (Fig. 5).

To date, 3 proteins that form complexes with CPT1C and are regulated by malonyl-CoA have been described:

- SAC1 is a phosphatidylinositol (PI4P) phosphatase that regulates PI4P levels in the Golgi and is involved in cholesterol and sphingolipid transport from the trans-Golgi network (TGN) to the plasma membrane. SAC1 is also a negative regulator of the secretory pathway [48]. Dysregulation of SAC1 and Golgi PI4P levels, which features in many tumors, is often associated with the release of proteins that remodel the extracellular matrix, promoting angiogenesis and cell motility, and is also associated with poor tumor prognosis [49, 50]. Our group has demonstrated that CPT1C downregulates SAC1 activity and increases PI4P levels in the ER-TGN contact sites depending on malonyl-CoA [16]. In this way, CPT1C may drive lipid membrane remodeling and favor the secretory pathway, therefore enhancing cell migration and invasion (Fig. 5).

Other interactors have been described for CPT1C, including the hydrolase ABHD12 [60]. ABHD12 silencing suppresses breast cancer cell proliferation, migration, and invasion [61]. Therefore, even though we still do not know how CPT1C regulates ABHD12 activity, we cannot rule out a role for the CPT1C-ABHD12 complex in cancer cell progression.

In this context, how can we explain the observation that CPT1C expression modulates FAO? Although it may well be through the interaction with ABHD6, as explained above, we propose 2 additional putative explanations. First, CPT1C may indirectly regulate CPT1A/B activity by locally buffering malonyl-CoA at mitochondria–ER contact sites. If so, CPT1C overexpression would sequester malonyl-CoA, leading to an increase in CPT1A/B activity and in FAO. Second, we cannot rule out that CPT1C at mitochondria–ER contact sites may interact with CPT1A/B or other mitochondrial proteins, thereby regulating their activity. Overall, we propose that CPT1C does not directly regulate FAO by mediating FA entry to mitochondria, but indirectly, whether by buffering malonyl-CoA locally at mitochondria–ER contact sites, or by interacting with other proteins involved in mitochondrial function (Fig. 5).

In summary, we are of the opinion that CPT1C in cancer cells behaves as a nutrient sensor that facilitates tumor progression and metabolic adaptation to environmental stressors through regulation of SAC1, protrudin, ABHD6, and possibly other proteins (Fig. 5).

## Conclusions and future perspectives

CPT1C is an ER transmembrane protein with inefficient catalytic activity, and as such, is not able to directly mediate FA transport to mitochondria for FAO. Instead, CPT1C acts as a malonyl-CoA sensor that, in regulating the function of other proteins such as SAC1, ABHD6, and protrudin, also regulates key cancer cell processes such as lipid metabolism, mitochondrial function, secretory pathway, and protrusion growth. Since malonyl-CoA levels fluctuate depending on nutrient availability, CPT1C could be said to be a nutrient sensor involved in metabolic reprograming of cancer cells to be able to adapt to constantly changing conditions in the tumor microenvironment.

In relation to FAO, since our results show that CPT1C is present at mitochondria–ER contact sites, we hypothesize that CPT1C may locally buffer malonyl-CoA and indirectly regulate CPT1A/B activity and FAO. Identifying novel mitochondrial proteins that interact with CPT1C would throw light on the wide spectrum of CPT1C effects on mitochondrial function.

Interestingly, in metabolism, neurons and cancer cells are similar in that they do not usually rely on fats for energy fuel. In neurons, lipids are reserved for the constant membrane remodeling that takes place during spinogenesis, synaptic plasticity, and interconnectivity; in cancer cells, lipids are reserved for continuous membrane synthesis during cell division and growth. Therefore, it is not unreasonable to think that cancer cells may take advantage of the neuronal protein CPT1C for their own benefit, i.e., to act as a nutritional sensor that regulates lipid metabolism in such a restricted context.

Future research should deepen understanding of the CPT1C-regulated molecular pathways that drive cell metabolic reprogramming. How does CPT1C drive survival in conditions of hypoxia and nutrient depletion? Which CPT1C-interacting proteins are involved? More specifically, how does CPT1C regulate mitochondrial function? Is it only by buffering the malonyl-CoA, or does it regulate the function of a specific mitochondrial protein? Are high CPT1C-expressing cancers more dependent on lipids as fuel? A better understanding of CPT1C downstream pathways would undoubtedly help us draw the complex and dynamic picture of cancer cell metabolic plasticity.

An important issue that needs to be addressed in the near future is the search for drugs that target CPT1C, and so prevent metabolic adaptation of cancer cells to hypoxia or nutrient depletion, as supportive treatment to conventional therapy. In xenograft mouse models, CPT1C depletion has been shown to reduce tumor growth and development, and, in several types of human tumors, low CPT1C expression is associated with increased overall survival. Because CPT1C is expressed only in the brain, while CPT1A and CPT1B are widely expressed in peripheral tissues, a promising anticancer strategy—as suggested by the group of Tak W. Mak [23, 37]—is the development of small drugs or sh-RNA-carrying nanomedicines that specifically target CPT1C but are unable to penetrate the blood–brain–barrier [23, 37]. Since CPT1C shows high homology with canonical isoforms, it is essential to understand small conformational variations between isoforms that may be druggable. Unfortunately, CPT1 protein structure predictions as provided by the AlphaFold database are not sufficiently accurate to reveal those small variations. Therefore, a prerequisite for designing new drugs targeting CPT1C is to elucidate the crystal structure of those proteins.

In summary, increased CPT1C expression favors the survival of cancer cells under metabolic stress. Moreover, it protects cancer cells from senescence and promotes their proliferation, migration, and invasion. CPT1C has been proposed as a prognostic marker for different human tumor types, and, as such, would contribute to better decision-making and more accurate follow-up of tumor treatment. Further insights to the molecular role of CPT1C in tumor cells would lead to a better understanding of the metabolic reprogramming undergone by cancer cells in response to environmental challenges, and should result in the design of more effective therapeutic strategies.

## Supplementary information

Supplemental data

## Data Availability

All data generated or analyzed during this study are included in this published article
