# Supplementary figures and images for: To be or not to be a fat burner, that is the question for cpt1c in cancer cells
Source: Cell Death Dis. 2023 Jan 24;14(1):57. doi: 10.1038/s41419-023-05599-1 (PMC9873675; doi:10.1038/s41419-023-05599-1)

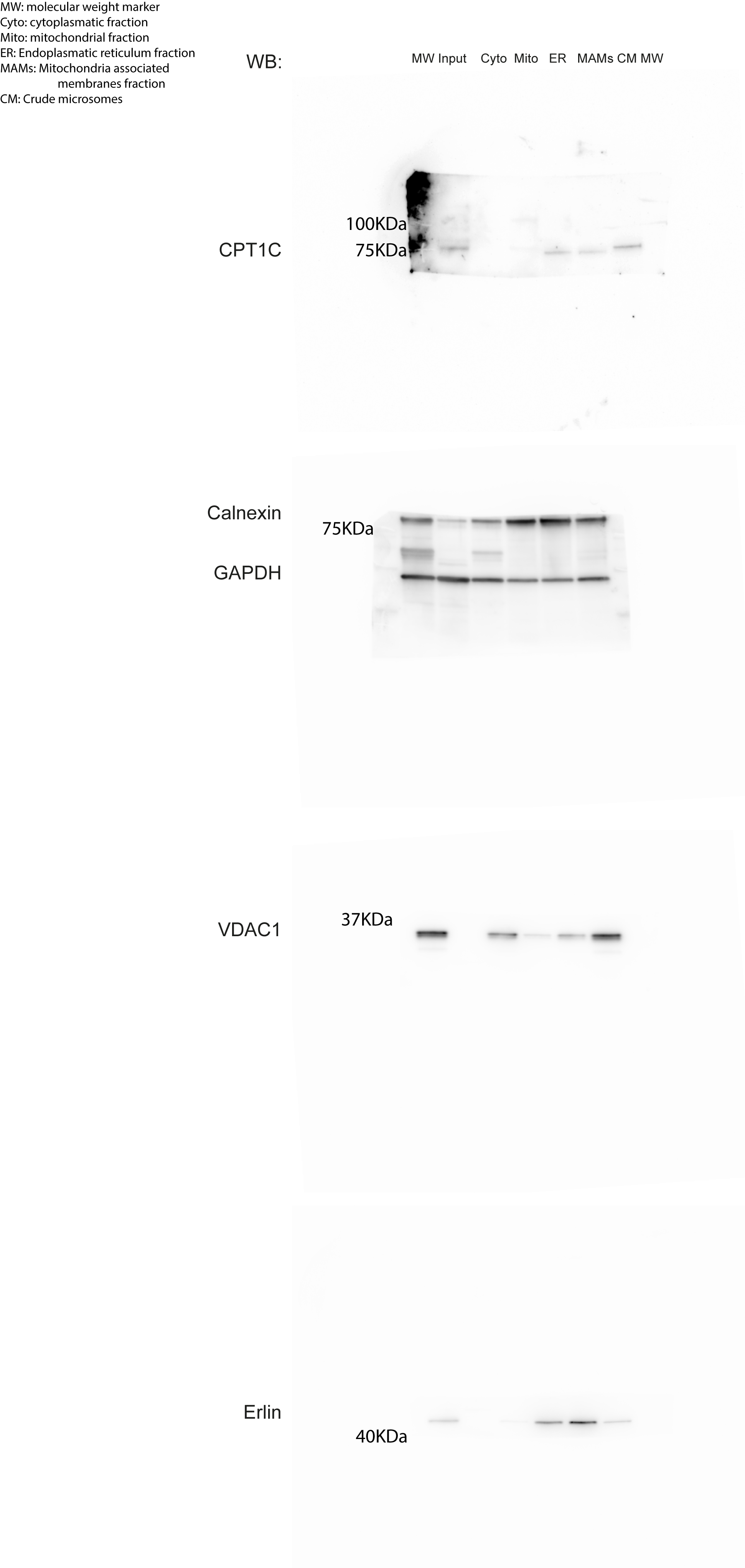

Supplement: Supplementary file 1 — Supplemental data [file 41419_2023_5599_MOESM1_ESM.tif]
